# Acidic pH Modulates Headgroup Orientation and Packing in Bis(monoacylglycero)phosphate Bilayers

**DOI:** 10.1021/acsphyschemau.5c00150

**Published:** 2026-03-10

**Authors:** Tayana Mazin Tsubone, Pedro Nunes de Oliveira Junior, Gustavo Scanavachi, Vinicius Firmino dos Santos, Antonio Rodrigues da Cunha, Ana Paula Ramos, Thereza A. Soares, Rosangela Itri

**Affiliations:** † Departamento de Física Aplicada, Instituto de Física, 28133 Universidade de São Paulo, São Paulo, SP 05508090, Brazil; ‡ Instituto de Química, Universidade Federal de Uberlândia, Uberlândia, MG 38400-902, Brazil; § Department of Pediatrics, Harvard Medical School, Boston, Massachusetts 02115, United States; ∥ Program in Cellular and Molecular Medicine (PCMM), Boston Children’s Hospital, Boston, Massachusetts 02115, United States; ⊥ Department of Chemistry, Faculty of Philosophy, Science, and Letters, University of São Paulo, Ribeirão Preto, SP 14040-901, Brazil; # Centro de Ciências de Balsas, 37892 Universidade Federal do Maranhão, Balsas, Maranhão 65800-000, Brazil; g Hylleraas Centre for Quantum Molecular Sciences, University of Oslo, Oslo 0315, Norway

**Keywords:** bis(monoacylglycero)phosphate, lipid bilayer, pH impact, lipid packing, molecular orientation

## Abstract

Bis­(monoacylglycero)­phosphate (BMP) possesses an atypical headgroup structure, and its naturally occurring 2,2′-isomer rapidly rearranges to the more stable 3,3′-form in aqueous buffers. Because the BMP is a major component of endosomal and lysosomal membranes, understanding its intrinsic physicochemical behavior is broadly relevant to the organization of acidic intracellular interfaces. To quantify how acidity modulates the physicochemical organization of 3,3′-BMP membranes, we characterized model bilayers across a controlled pH range by using electrophoretic mobility, fluorescence spectroscopy, optical microscopy, small-angle X-ray scattering (SAXS), dynamic light scattering (DLS), and atomistic molecular dynamics (MD) simulations. Below pH 5, BMP vesicles show a marked decrease in surface charge, a reduction in area per lipid, and diminished hydration at the polar–apolar interface. Simulations reveal that protonation enables transient flipping of the phosphate group toward the hydrophobic core, generating a pH-dependent coexistence of headgroup orientations absent in the deprotonated state. Experimentally, these changes manifest as reduced vesicle sizes and the emergence of multilamellar protrusions and locally folded membrane regions. The combined results demonstrate how small variations in the BMP charge state generate amplified structural responses at the bilayer level, establishing a molecular mechanism by which protonation governs packing, hydration, and curvature in BMP-rich membranes.

## Graphical abstract

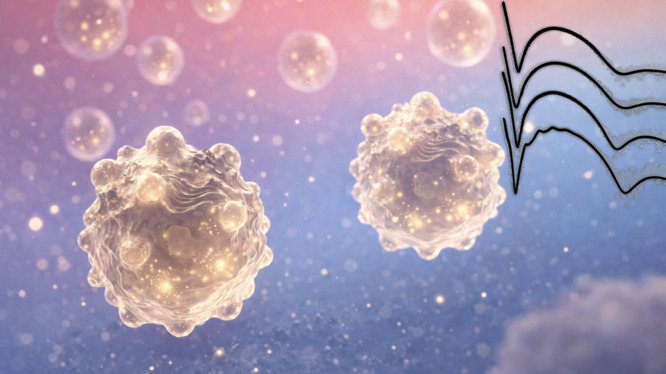

## Introduction

The phospholipid bis­(monoacylglycero)­phosphate (BMP) is an anionic lipid at physiological pH, carrying a single negative charge on its phosphate group (Figure 1). Unlike most glycerophospholipids, which contain a typical *sn*-3-glycerophosphate backbone, BMP consists of two glycerol moieties that can adopt distinct stereochemical configurations, making it a structural isomer of the anionic lipid phosphatidylglycerol,^1–5^ where each glycerol unit bears a single acyl chain (Figure 1). Commercially available BMP carries its acyl chains in the 3,3′-positions, a configuration that is more stable in vitro than the naturally occurring 2,2′-linked form, in which the phosphate-bearing glycerol occupies the 2,2′-positions.^6–8^ The 2,2′-dioleoyl BMP isoform is the predominant species in cells (≈90%), accounting for roughly 15 mol % of total endosomal/lysosomal lipids and up to ∼70 mol % of their internal membrane phospholipids. Its unusual stereochemistry and molecular geometry have been linked to characteristic physicochemical behaviors, particularly under acidic conditions.^3^ Prior studies show that BMP can promote multilamellar organization or highly curved vesicular structures in a pH-dependent manner, suggesting a role in modulating membrane curvature and packing stress.^3,6,9^ However, probing the membrane properties of endosomes and lysosomes in intact cells remains challenging due to the continuous maturation, remodeling, fusion, and fission of these organelles. Consequently, simplified model bilayers offer a powerful approach to elucidate the physicochemical features of BMP and their implications for membrane organization.^10^

**Figure 1:**
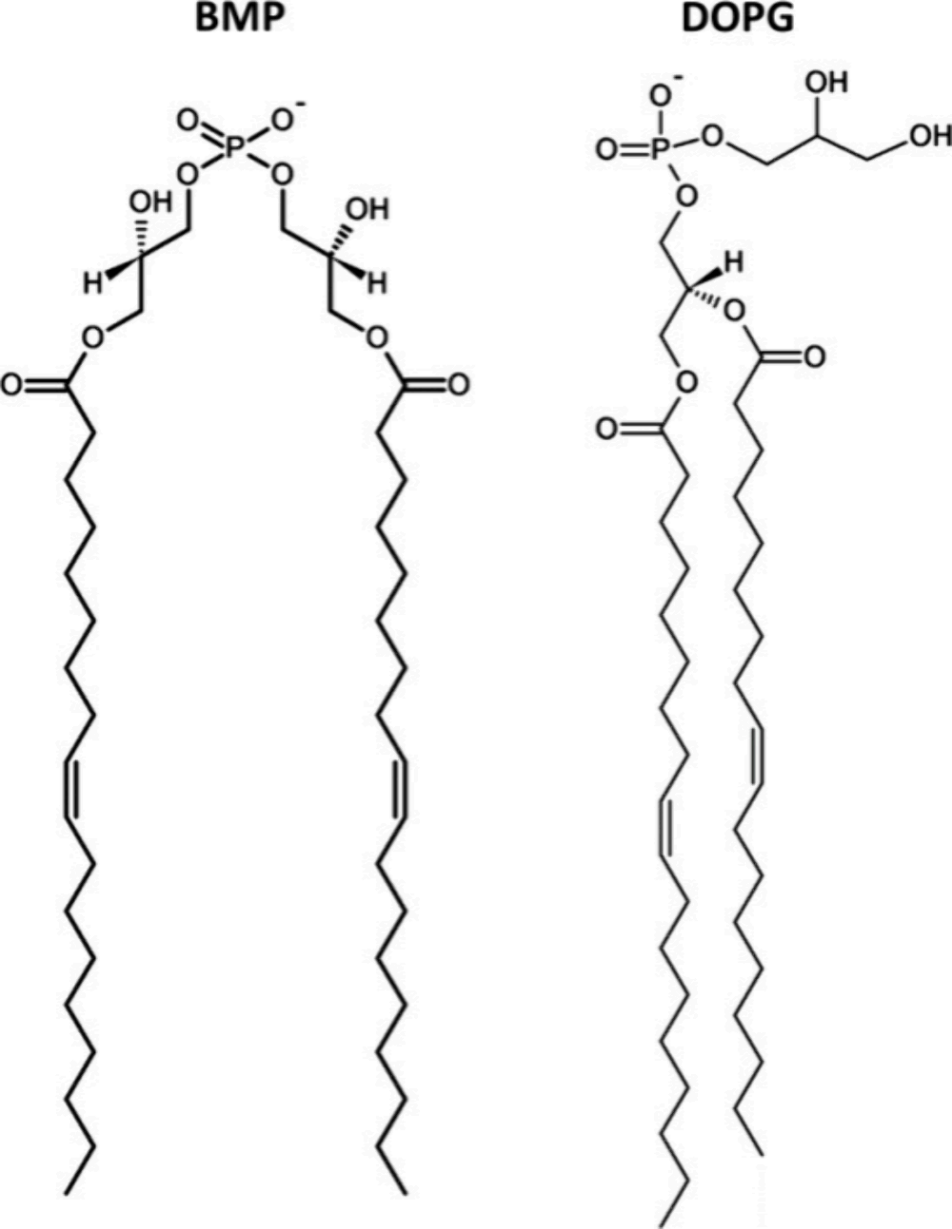
Structures of the commercially available 3,3′-BMP (bis­(monoacylglycero)­phosphate) and DOPG (1,2-dioleoyl-*sn*-glycero-3-phospho-(1′-rac-glycerol)) at physiological pH 7.4.

Herein, we investigated how environmental pH modulates the structure and properties of lipid bilayers composed exclusively of 3,3′-BMP (Figure 1), by combining molecular dynamics (MD) simulations with a suite of experimental techniques, including small-angle X-ray scattering (SAXS), dynamic light scattering (DLS), electrophoretic mobility, fluorescence spectroscopy applied to large vesicles (LUVs), and optical microscopy to giant unilamellar vesicles (GUVs). Results were compared with those obtained for bilayers formed by the anionic lipid 1,2-dioleoyl-*sn*-glycero-3-phospho-(1′-rac-glycerol) (DOPG), a structural isomer of 3,3′-BMP (Figure 1). Under acidic conditions, we observed pronounced changes in the membrane surface charge, curvature, hydration, and lipid packing. Notably, BMP GUVs at pH 4.5 displayed striking morphological features including numerous membrane protrusions and an interconnected multilayered network suggestive of local membrane folding. MD simulations further revealed conformational changes accessible to 3,3′-BMP at low pH, and additional simulations of the natural 2,2′-dioleoyl BMP isomer (data not shown) indicate that both stereoisomers exhibit similar protonation-dependent conformational behavior at the molecular level.

## Experimental Section

### Materials

The lipids DOPG (1,2-dioleoyl-*sn*-glycero-3-phospho-(1′-rac-glycerol) sodium salt) (CAS Number 67254–28–8) and BMP (bis­(monooleoylglycero)­phosphate (*S,R*-isomer) ammonium salt) (CAS Number 326495–20–9) were purchased from Avanti Polar Lipids and used without further purification. The 100 nm polycarbonate extrusion membranes and filter supports were also acquired from Avanti Polar Lipids. 4-(2-Hydroxyethyl)-1-piperazineethanesulfonic acid (HEPES), sodium acetate, and Triton X-100 were obtained from Sigma-Aldrich. Milli-Q water was employed under all circumstances.

### Giant Unilamellar Vesicles (GUVs)

GUVs were prepared by a gel-assisted method.^11^ Briefly, 60 μL of 5% poly­(vinyl alcohol) (PVA) solution in water was spread on the surface of the microscope coverslip and dried in an oven at 70 °C for 60 min. Next, 10 μL of a 2 mM lipid (DOPG or BMP) solution in chloroform was spread onto the surface of glasses coated with 5% PVA previously dried. The sucrose and glucose solutions were prepared at the desired pH, 5 mM HEPES buffer at pH 7.4, or 5 mM sodium acetate buffer at pH 4.5. Sucrose and glucose solutions were also prepared in Britton–Robinson buffer to control pH between 2 and 12, keeping the same ions. Britton–Robinson buffer was prepared by mixing 0.04 M phosphoric acid, 0.04 M boric acid, and 0.04 M acetic acid as previously described.^12^ A chamber was built up with a Teflon spacer and filled with 0.2 M sucrose solution (at the required pH) and incubated for 1 h at room temperature. Then, the GUV suspension was diluted in a glucose solution (dissolved in the same buffer and pH as those of sucrose solution). The osmolarity was controlled by means of an osmometer (Osmomat Gonotec 030). GUVs were visualized by phase contrast mode using an inverted optical microscope (Axiovert 200, Carl Zeiss) equipped with Ph2 objective 63×, at a temperature of 23 °C. Images were registered with an Axiocam HSm digital camera (Carl Zeiss) and analyzed using *ImageJ* and a homemade C++ code using OpenCV library^13^ to perform automated GUV detection, tracking, and further quantification.

### Evaluation of the Average Size of GUVs and Heterogeneity

The GUV diameters were measured from images obtained under phase contrast microscopy using *ImageJ* software and a homemade C++ code using the OpenCV library for GUV detection and diameter (*D*) measurements. Briefly, the GUV diameter was obtained by applying a Gaussian blur filter in the raw image for better edge detection followed by finding the possible circle centers and the best radius for each candidate center, which is optimized by a minimization algorithm resulting in an accurate circle fitting and diameter measurement. The center of each GUV was defined as the center of the best-fit circle obtained from this procedure.

Some GUVs from our data revealed membrane protrusion and a net of interconnected multilamellar structures over the membrane surface, which we named as “heterogeneity” in the vesicle. In order to quantify it, the heterogeneity, *H*, of each GUV was determined according to eq 1:

1 $$H=\Sigma_{\theta =0^{{}^{\circ}}}^{179^{{}^{\circ}}}\left(\frac{1}{r}\Sigma_{i=1}^{N_{r}}\left|I_{i}\left(\theta \right)-I_{-i}\left(\theta \right)\right|\right)$$

where *r* is the radius of the GUV (*r* = *D*/2); *N*_r_ is the number of symmetric pixel pairs (*i*, −*i*) sampled along a radial line from the GUV center to the membrane, which corresponds to the vesicle radius expressed in pixels; *I*_*i*_ is the pixel intensity of the pixel *i* and *I*_–*i*_ is the pixel intensity of the pixel −*i*, which is at the symmetric opposite distance from the GUV center as the *i* pixel position. The absolute value |(*I*_*i*_ – *I*_–*i*_)| gives the pixel intensity difference between two pixels that are equidistant from the center of the GUVs and on the same line that goes through the center. This operation was performed from the GUV center to the radius and normalized by it so that the resulting value represents an average intensity difference per unit radial length, thereby removing size-dependent effects and enabling comparison between GUVs with different sizes. Further, we calculated from 0° to 179° to obtain the heterogeneity for all angles. Typical analysis contained around 15–25 GUVs.

### Small-Angle X-ray Scattering (SAXS) Measurements

Liposomes of 10 mM BMP or DOPG were prepared by hydration of lipid film with buffer at the appropriate pH. A 10 mM sodium acetate buffer was used at pH 4.5 and a 10 mM HEPES buffer at pH 7.4. Then, samples were passed 21 times through 100 nm extrusion membrane pores, at 25 °C. Samples were kept at 4 °C prior to the SAXS experiments that were carried out at the National Laboratory of Synchrotron Light (LNLS, Campinas, Brazil). The scattering vector modulus *q* = 4π sin θ_SAXS_/λ, with 2θ_SAXS_ being the scattering angle and λ the X-ray wavelength of 1.548 Å, ranged from 0.013 to 0.50 Å^–1^. Samples were placed in a 1 mm path length mica holder, and the exposure time was 300 s. The experimental scattering intensity, *I*(*q*), was analyzed considering the scattering from a large vesicle, where the lipid bilayer thickness is much smaller than its size, described as a product of the form factor, *P*(*q*), and the structure factor, *S*(*q*), as:^14^

2 $$I\left(q\right)=\kappa P\left(q\right)S\left(q\right)=\kappa \frac{2\pi A}{q^{2}}P_{t}\left(q\right)S\left(q\right)=\kappa \frac{2\pi A}{q^{2}}P_{t}\left(q\right)\left[u+\left(1-u\right)S_{MCT}\left(q\right)\right]$$

with κ being the scaling factor, *A* the area of the basal plane, and *P*_*t*_(*q*) the form factor of the lipid bilayer cross section perpendicular to the *A* plane. *S*_MCT_(*q*) is the structure factor according to the modified Caillé theory;^15^ *u* is the fraction of uncorrelated bilayers; and (1 – *u*) is the complementary fraction of correlated bilayers, whose correlations are described by *S*_MCT_(*q*). *P*_*t*_(*q*) was modeled as the Fourier transform of the scattering density here represented by step functions of three different electron densities with respect to the aqueous solution electron density.^15^ DOPG and BMP SAXS curves at pH 7.4 are typical of unilamellar vesicles such that we consider *S*(*q*) = 1 (*u* = 1) in eq 2 in the data analysis. The corresponding fitting parameters associated with the form factors *P*_*t*_(*q*) are presented in Supporting Information, Table S1. At pH 4.5, SAXS data presented features of multilamellar structures coexisting with unilamellar vesicles (see Results). In this case, the *S*_MCT_(*q*) function was modeled considering the modified Caillé theory (MCT)^16^ as described elsewhere,^15^ where a main parameter is the Caillé parameter, η_Caillé_, related to the membrane packing/order and membrane fluctuations. All fittings to the experimental SAXS data were done by using the Genfit software.^17^

### Zeta-Potential Measurements from Liposomes

Zeta potential (ζ) from 0.5 mM BMP or 0.5 mM DOPG liposomes was prepared by hydration of lipid film in Milli-Q water and passed through extrusion membrane pores of 100 nm. Then, ζ of the samples was measured in a Zetasizer Nano ZS (Malvern Instruments) calculated from the electrophoretic mobility based on the Helmholtz–Smoluchowski equation.^18^ The curves of ζ versus pH were also obtained. For this, 0.1 M HCl was added until the sample stabilized at pH 2 (starting point), and then automatic titration (Malvern instruments) with 0.1 M NaOH was performed to reach the desired pH value. Measurements were repeated at least three times at 25 °C.

### Titration Curves

Titrant solutions were prepared from 0.010 M HCl and added to a well-stirred aqueous suspension of lipid (0.5 mM BMP or 0.5 mM DOPG) in aliquots of 10–40 μL. Dispersion of 1.5 μM lipids was prepared by hydration of lipid film in Mili-Q water. Manual reading of the pH was taken by periodically interrupting (each 2 min) the titration to verify the pH value. The response of pH electrode was checked before starting titration in standard buffer solutions with pH of 7.00 and 4.01 at 25 °C. All titrations were done on the lipid suspension at room temperature (23 ± 1 °C).

### Fluorescence Measurements of Laurdan Generalized Polarization (GP)

Laurdan is a fluorescent molecule that exhibits a shift in its emission spectrum depending on the polarity of its microenvironment.^19,20^ It has been reported that Laurdan is located in the membrane at the glycerol backbone.^21,22^ Therefore, Laurdan senses the polarity of the environment surrounding the glycerol backbone of the lipid molecules. Because water penetration in the bilayer correlates with lipid packing and membrane fluidity,^21,22^ Laurdan is largely used to infer changes in hydration/dehydration (polarity) and lipid order. Polarity changes can be quantified by calculating the generalized polarization (GP) defined as eq 3:^20^

3 $$GP=\frac{I_{440}-I_{490}}{I_{440}+I_{490}}$$

where *I*_440_ and *I*_490_ are the emission intensities at 440 and 490 nm, respectively. GP values can vary from 1 (no polar solvent effect) to −1 (completely exposed to water).^20^

Laurdan was included here on each lipid film under a proportion of 1:200 (Laurdan/lipid). Then, lipid films (BMP or DOPG) were hydrated in 0.5 mM HEPES, 0.5 mM EDTA, pH 8.0, to form multilamellar vesicles (MLVs). The pH of samples was gradually lowered by adding aliquots of 100 mM HCl, and Laurdan fluorescence emission spectra (from 370 to 600 nm) were registered at distinct pH values: 8.1, 7.5, 7.0, 6.5, 5.8, 4.2, 2.9, and 2.1. Laurdan’s excitation wavelength was 355 nm. All measurements were carried out at a lipid concentration of 0.1 mM and recorded at 25 °C. Three independent samples were measured for each lipid system (BMP or DOPG).

### Computational Simulations

Molecular dynamics (MD) simulations were carried out for bilayers composed of 256 lipid units of BMP at neutral (BMPD) and low (BMPP) pH and DOPG lipids. The initial configurations of the three systems were generated through the CHARMM-GUI Web server,^23,24^ and the CHARMM36 force field was used^25^ together with the TIP3P water model.^26^ The BMP topologies and atomic parameters at low and neutral pH are presented in the Supporting Information (Figure S1). Simulated systems were neutralized by the addition of Na^+^ and Cl^–^ to reproduce salt concentrations of 150 mM.

Energy minimization was performed for all of the systems until a mean force of less than 1000 kJ mol^–1^.nm^–1^ was achieved. The equilibration phase was performed for 500 ps in the NVT ensemble and subsequently in the NPT ensemble at 303.15 K and a time step of 1 ps. The production phase was carried out in the NPT ensemble with a time step of 2 fs. Initial velocities were taken from a Maxwell distribution at 300 K and 1 atm. Bond lengths within the solute were constrained using the LINCS algorithm.^27^ The temperatures of the solute and solvent were controlled by separately coupling them to a thermostat. The Berendsen thermostat with a relaxation step of 0.1 ps^27^ was used during the equilibration phase and switched to the Nose–Hoover thermostat with a relaxation time of 1.0 ps during the production phase and temperature of 303.15 K.^28^ The pressure was maintained at 1 atm with the Berendsen barostat and a relaxation time of 1.0 ps^28^ and the Parrinello–Rahman barostat with a relaxation time of 5.0 ps during the equilibration and production phases, respectively.^29^ The pressure coupling algorithm was used with semi-isotropic coordinate scaling and an isothermal compressibility of 4.5 × 10^–5^ (kJ mol^–1^ nm^–3^)^−1^. Nonbonded interactions were treated with a cutoff of 1.4 nm. Long-range electrostatic interactions were treated with the Particle Mesh Ewald summation, and charges were projected onto a 0.16 nm grid using a cubic interpolation for the calculation in reciprocal space.^30^ The systems were equilibrated for 200 ns when the average area per lipid (*A*_L_) values reached convergence. The production phase was carried out for 300 ns. The GROMACS 2021.3 software was used for the MD simulation and some of the analysis.^31^ The SuAVE software was also used for analyses of properties dependent on the membranes curvature such as area per lipid *A*_L_, volume per lipid (*V*_L_), and membrane thickness (*D*_HH_).^32,33^ The analysis of the conformational populations of the flipped-out and flipped-in states from MD simulations of deprotonated BMPD (neutral pH) and protonated BMPP (acidic pH) was performed, as presented in Figure 2.

**Figure 2:**
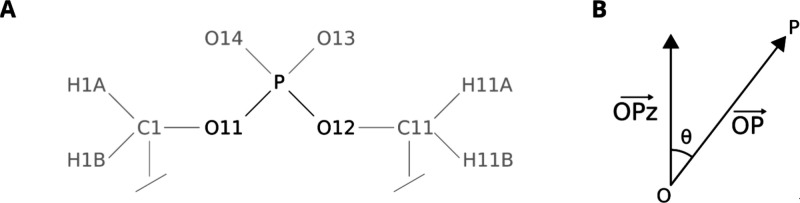
(a) Head of the lipid, with atoms used in the analysis in black. (b) Definition of the angle between the O–P bond and the *z*-axis of the simulation box.

The orientation of the phosphate group can be determined by calculating the angles of the O11–P and the O12–P bonds with the *z* axis of the simulation box. Thus, if the O11–P bond is perfectly parallel to the *z* axis of the box (P pointing up), the resulting angle is 0°. If the bond is antiparallel to the *z* axis (P pointing down), the resulting angle between them is 180°. The same is true for the oxidized adduct, the O12–P bond. Taking the cosine of the angle θ, calculated values are −1, for P pointing down, and 1, for P pointing up. This can be easily calculated because GROMACS can give the necessary quantities. If we use the *z* component of the O–P bond instead of using the *z* axis of the box, as shown in Figure 2b, the cosine of the angle is then given by the equation:

4 $$\cos\left(\theta \right)=\frac{\left||O{\mathrm{P⃗}}_{z}|\right|}{\left||O\mathrm{P⃗}|\right|}$$

where $||\overset{⃗}{OP}||$ is the length of the OP bond and $||\overset{⇀}{OP_{z}}||$ is the length of the *z* component of the OP vector defined in Figure 2b. These two quantities can be easily calculated using the routine *gmx distance* in the GROMACS software. If the value of cos­(θ) is between 0 and 1 for both O11–P and O12–P, the phosphate is flipped up. If the values are between −1 and 0, the phosphate is flipped down. To make Figure 8a and 8d, the O11–P bond was defined as bond 1 and O12–P as bond 2. The analysis was made using all of the lipids of the upper leaflet of the membrane.

## Results and Discussion

### BMP Vesicle Morphology is pH-Dependent

In order to better understand how the pH environment can affect the morphological behavior of 3,3′-BMP-containing membranes, here we investigate a minimal system represented by giant vesicles composed only of BMP dispersed in a Britton–Robinson buffer solution (see Materials). In contrast to the smooth membrane surface at pH 7.4, phase contrast microscopy images revealed membrane protrusion at pH 4.5 and a net of interconnected multilamellar structures over the membrane surface (Figure 3A). Of note, GUVs composed of DOPG, an isomer of BMP (see Figure 1), do not present the same pattern in either acid or neutral medium (Figure 3A).

**Figure 3:**
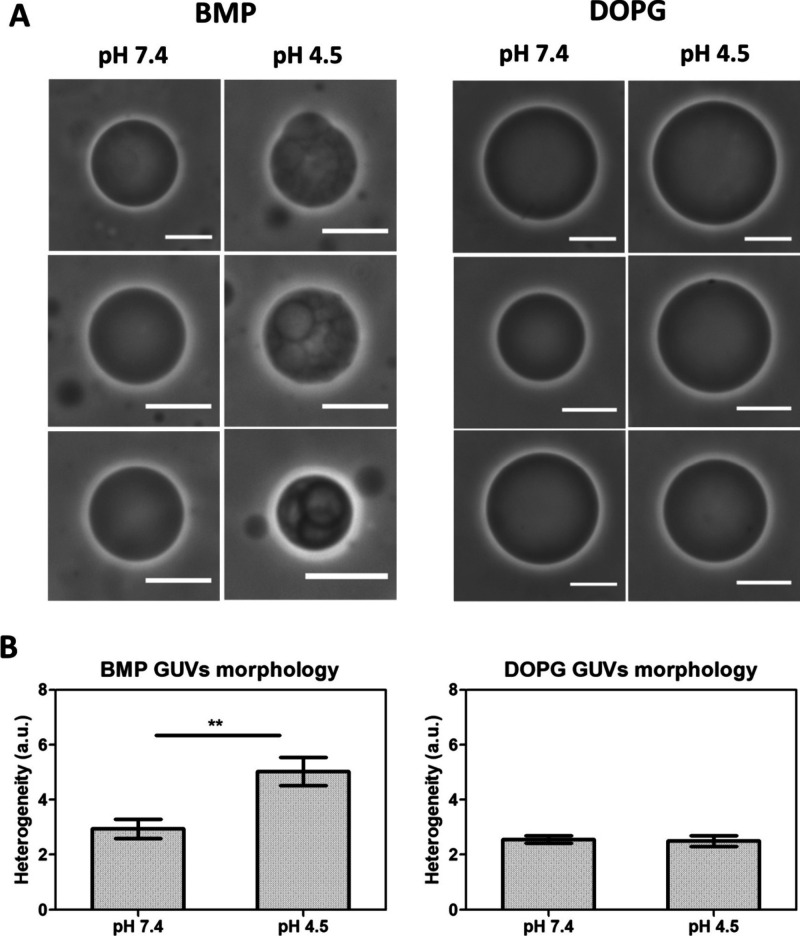
(A) Phase contrast microscopy of 3 representative images of GUVs composed of either 3,3′-BMP or DOPG at pH 7.4 and pH 4.5. Scale bars correspond to 10 μm. Both pH values were prepared in Britton–Robinson buffer. (B) Heterogeneity of spherical vesicles. Bars represent means ± standard error of the mean (SEM) and ***p < 0.0001.

We quantified the heterogeneity over the vesicle surface by using homemade software developed by our group (see Materials). Indeed, the heterogeneity factor for GUVs of DOPG in both pH values was close to zero (smaller than 5), while GUVs of BMP at pH 4.5 presented heterogeneity values higher than 5 (Figure 3B).

It is important to mention that this distinct behavior of BMP membranes in acidic media is not related to different ions from buffers. Experiments using different buffers (sodium acetate for pH 4.5 and HEPES for pH 7.4) showed similar features in vesicle morphology (Figure S2).

Interestingly, SAXS data from BMP LUVs (Figure 4) evidenced the appearance of small peaks (1st at *q* ∼ 0.30 nm^–1^, see Figure S3 – black arrows) over the lipid bilayer scattering profile at pH 4.5 (black dots) in contrast to the SAXS curves from BMP LUVs at pH 7.4. Such small peaks may be correlated to some local arrangement of the folded membrane as observed on GUVs images at pH 4.5 (Figure 3A). On the other hand, SAXS data from DOPG LUVs at pH 4.5 showed only one slight bump around *q* ∼ 0.24 nm^–1^ (see Figure S3 – black arrows), not present at pH 7.4. Such a bump at pH 4.5 may suggest the presence of DOPG interbilayer weak interaction (Figure 4).

**Figure 4:**
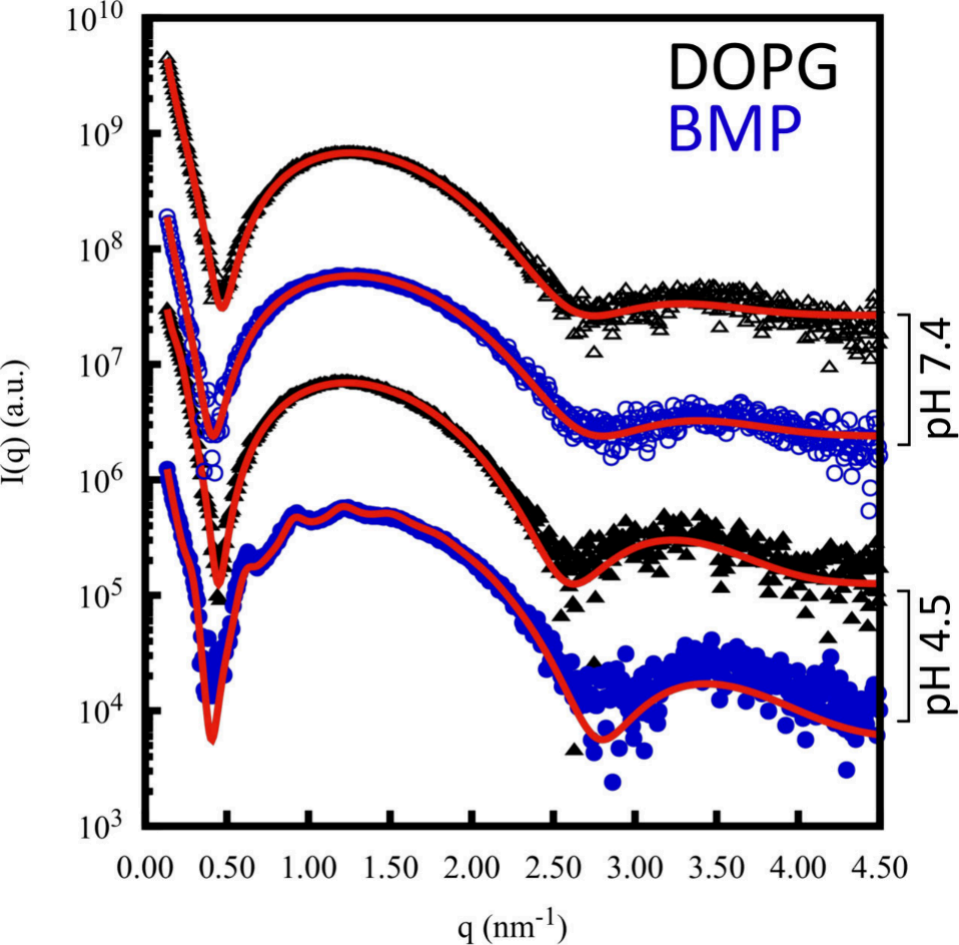
SAXS curves of LUVs composed of 10 mM DOPG (black) and 10 mM BMP (blue) at pH 7.4 (5 mM HEPES buffer, open symbols) and at pH 4.5 (5 mM acetate buffer, full symbols) at 23 °C. The best fit for each experimental curve is depicted in a continuous red line. The fitting parameters are displayed in Table S1. The curves are shifted for better visualization.

The analysis of the SAXS curves by fitting the curves (eq 2) to the experimental data revealed that the parameters related to the form factors, *P*_*t*_(*q*), of BMP and DOPG lipid bilayers, at both pH values, did not show any significant difference (Table S1). Likewise, the corresponding electron density profiles did not present any significant difference (Figure 7), consistent with the density profiles obtained from the MD simulations (see below). Of note, the obtained parameters for the DOPG lipid bilayer are in good agreement with results published elsewhere.^34^ With respect to the analysis performed at pH 4.5 regarding the stacking of lipid bilayers via the *S*(*q*) function (Figure S4), the results point out a stacking of 2–3 bilayers for both BMP and DOPG, but η_Caillé_ is quite small for BMP (Table S1), implying low membrane fluctuations. Further, the interlamellar repetition distances are circa 21 and 27 nm for BMP and DOPG bilayers, respectively (Table S1).

### Protonation of BMP Influences Vesicle Size, Surface Charge, Hydration, and Lipid Packing

Previous reports indicate that membranes containing 2,2′-BMP can adopt diverse morphologies, a behavior often attributed to the distinctive stereochemistry and molecular geometry of this lipid.^35^ In our simplified systems, these same intrinsic features appear to govern membrane curvature and size distribution by modulating the balance among headgroup charge, hydration, and acyl chain packing. Then, the size of 100 nm extruded 3,3′-BMP and DOPG vesicles was characterized by DLS. The results (Figure S5) evidenced that DOPG and BMP at pH 7.4 form LUVs of circa 75 and 68 nm, respectively (considering the values of the greatest frequencies in the sizes), while such frequency of sizes is slightly displaced to 68 nm for DOPG and to a broader distribution of smaller vesicles centered on 55 nm for BMP LUVs at pH 4.5.

Concomitantly, GUVs displayed diameters smaller than 10 μm for BMP membranes in an acid medium, while other samples exhibited diameters larger than 15 μm at pH 7.4 (Figure S6). Therefore, these findings indicate that the BMP protonation state must play a role in the molecular packing/curvature probably through changes in the lipid geometrical shape. We will return to this point later in the text.

In the following, we propose that the protonation of BMP in the membrane leads to a partial charge neutralization. In an effort to prove this hypothesis, we evaluated the effect of pH on the ζ potential of LUVs and membrane lipid packing.

A pH-induced modification on the surface charge of LUVs was detected for both lipids (DOPG and BMP); however, the magnitude of the negative ζ-potential for BMP is lower than that for DOPG (Figure 5A). Note that at pH 3 BMP vesicles exhibit ζ-potential close to zero (Figure 5A), indicating that the p*K*_a_ of BMP lies near this pH. In fact, titration curves estimated a p*K*_a_ around 3.5 for BMP and DOPG liposomes (Figure S7). It agrees with apparent p*K*_a_ values of DOPG (3.1–3.5) already reported by other authors.^36–38^ It means that at pH 4.5 only part of the lipids (∼10%) are protonated (Figure S7).

**Figure 5:**
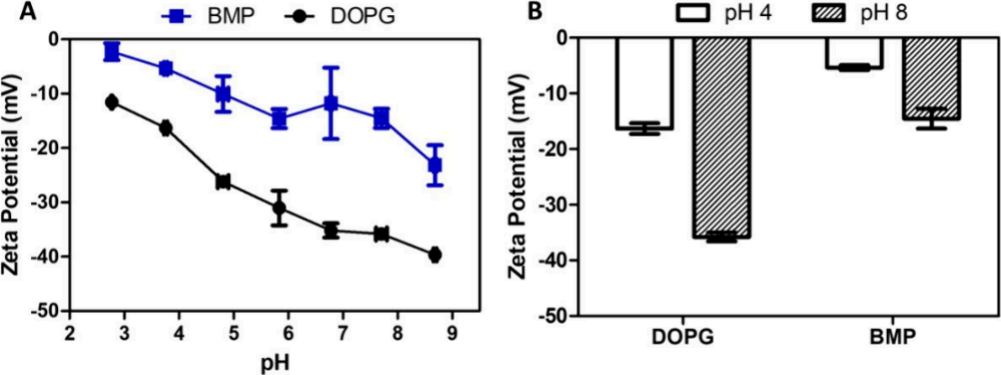
(A) ζ-potential (mV) of LUVs made of 0.5 mM BMP (blue line) and 0.5 mM DOPG (black line) as a function of pH, measured at 25 °C. Error bars correspond to ± standard deviation (SD). (B) ζ-Potential values specifically at pH 4 and pH 8 for both membranes BMP and DOPG. Bars correspond to mean value ± SD.

The ζ-potential at pH 4 for LUVs made of BMP is around −5 mV, while LUVs of DOPG present a value of about −16 mV (Figure 5B). Because ζ-potential values near neutrality (from ±0 to 10 mV) indicate diminished electrostatic repulsion among charged surfaces,^18^ the near-zero potential of BMP vesicles at pH < 5 reveals that partial protonation substantially reduces the surface charge density. This reduced repulsion favors closer headgroup association, promoting tighter lipid packing and decreasing overall colloidal stability.

Variations in the hydration properties at the polar/apolar interface were assessed by monitoring the fluorescence of the Laurdan probe incorporated in BMP and DOPG bilayers. It has been reported that Laurdan is located near the lipid glycerol backbone.^21,22^ Thus, it reports changes in the polarity surrounding the glycerol medium, and hence, it senses the lipid polar/apolar interface. Then, we incorporated Laurdan into BMP membranes and followed the fluorescence spectra under the gradually lowering pH of the MLV samples by adding aliquots of acid solution (100 mM HCl). Normalized emission spectra are displayed in Figure S8, and GP values are presented in Figure 6A. In order to compare the variations of the GP values, we calculated ΔGP for each different lipid system: the difference between the GP value and its initial value at pH 8.0 (Figure 6B). Figure 6A shows that all GP values obtained were lower than zero, indicating that membranes are in fluid phase regardless of their pH conditions.^39^ We observed that GP values remain constant between pH 6.0 and 8.0 for BMP vesicles, while below pH 5.0, GP values increase significantly from −0.39 to −0.02 (Figure 6A). On the other hand, only a slight increase in GP values for DOPG was observed (from −0.34 to −0.23), which is different from that found for BMP (from −0.39 to −0.02). Therefore, our results evidence a significant dehydration close to the glycerol backbone under phosphate group protonation, unlike the DOPG at acidic environment. This finding will be complemented by the molecular dynamic simulation results given below. Noteworthy, under dilution there are no significant changes in the Laurdan fluorescence spectra, such that GP values remain the same whatever the dilution (Figure S9).

**Figure 6:**
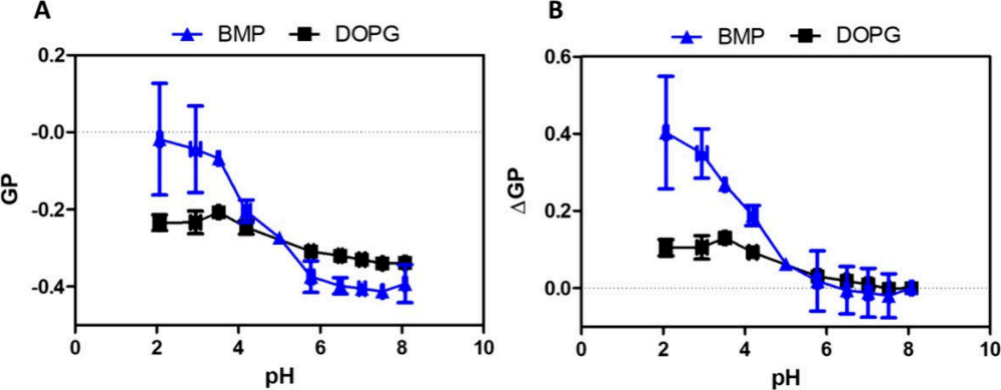
Effect of pH medium on membrane packing/hydration. (A) GP and (B) ΔGP values as a function of pH for BMP (▲) and DOPG (■) vesicles. All measurements were carried out at a composition of 1:200 Laurdan/lipid and were recorded at 25 °C. pH of the MLV samples was gradually lowered by adding aliquots of acid solution (100 mM HCl) in the external solution. ΔGP is the difference between the GP value and its initial value at pH 8. Error bars correspond to the ± SD.

We also determined the area occupied by lipid molecules at the air–liquid interface by means of Langmuir monolayers, using neutral and acid buffers as subphases (Figure S10). The mean area occupied per BMP molecule at surface pressure *p* = 30 mN/m (the value where the thermodynamic parameters of the monolayer and bilayer area equalized)^40,41^ decreases from 0.721 ± 0.003 nm^2^ at pH 7.4 to 0.659 ± 0.003 nm^2^ at pH 4.5 which represents a reduction of 9%. This result is a consequence of the reduced surface charge of BMP at lower pH values, which minimizes repulsion among lipid polar heads. The DOPG monolayer is similar to that previously reported.^42^ The mean area per molecule does not change as a function of pH; i.e., at surface pressure of 30 mN/m, DOPG monolayers presented an area per molecule of ≈0.762 ± 0.006 Å^2^ (Figure S10B). In order to provide a microscopic interpretation of the experimental observations, molecular dynamics simulations were carried out as follows.

### Computational Simulations

MD simulations were performed for BMP in the neutral (BMPD) and protonated (BMPP) states and for its structural isomer DOPG. Average structural properties such as area (*A*_L_) and volume (*V*_L_) per lipid and membrane thickness are compared for the three simulated systems (Table S2). The normalized density profiles obtained from the MD simulations are compared with those retrieved from SAXS data analysis (Figure 7). The density profiles obtained from MD simulations of the BMPD and DOPG membranes are very similar. However, the protonation of BMPP promotes two main changes in the membrane structure (Figure 7): there is a slight increase in the membrane thickness, *D*_HH_, concomitant with a decrease in the hydration of the phosphate moiety compared to both BMPD and DOPG (Figure 7). The increase of *D*_HH_ upon protonation of the BMP (from 3.37 to 3.68 nm) is consistent not only with the decrease in the area per lipid *A*_L_ from 0.758 to 0.651 nm^2^ (Table S2) but also with the increase in acyl chain orientational order as measured by deuterium order parameters *S*_CD_ (Figure S11). The decrease in *A*_L_ upon protonation of BMP was also observed in the Langmuir monolayer assays (Figure S10). Differences in the calculated and experimental values are expected as comparisons are made between experimental monolayers under a lateral pressure of *p* = 30 mN/m and simulated bilayers treated as infinite along the *xy*-plane.

**Figure 7:**
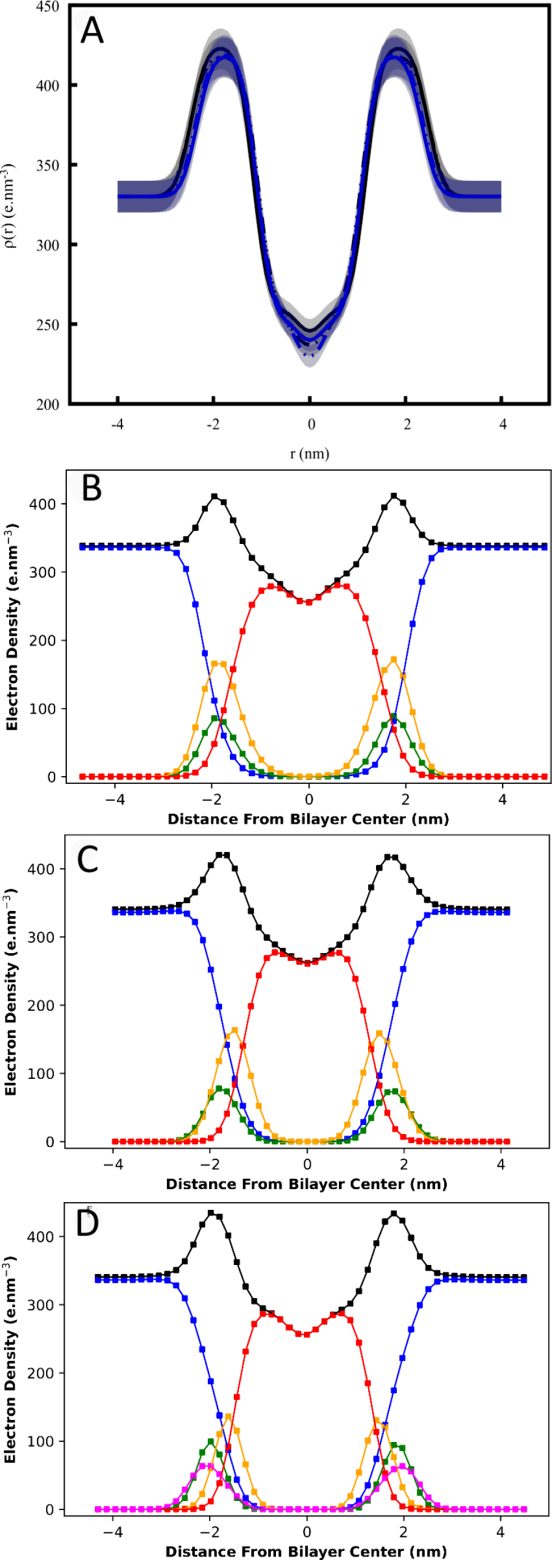
Electron density profiles were obtained from SAXS and MD simulations. (A) Electron density profile ρ­(*r*) calculated using the *P*_*t*_(*q*) parameters obtained through the fittings to the experimental SAXS curves for 10 mM DOPG (black) and 10 mM BMP (blue) at pH 7.4 (5 mM HEPES buffer, dashed line) and at pH 4.5 (5 mM acetate buffer, solid line) at 23 °C. The shade indicates the error as 3% for each ρ­(*r*). MD simulations of (B) BMPP, (C) BMPD, and (D) DOPG. Chemical groups are shown in green (phosphate), brown (glycerol), red (acyl chains), pink (glycerol derived from phosphatidylglycerol), and blue (water).

The decrease in hydration of the phosphate group of the protonated BMP can be linked to the flipping of the phosphate group, which results in two conformational states (Figure 8). In the flipped-out state, the phosphate group is positioned outward from the bilayer surface, in the bulk solvent (Figures 8A, 8C). In the flipped-in state, the phosphate group swings inward from the membrane surface into the hydrophobic core (Figures 8D, 8F). This structural transition positions the hydroxyl group between the two acyl chains, where it can form hydrogen bonds with the ester and carbonyl groups of these chains (Figure 8F). In fact, without the potential to form such interactions, the displacement of the hydroxyl group from the solvent into the hydrophobic region would be energetically unfavorable. The flipped-in conformational state is observed exclusively for the protonated BMP membrane, where it occurs in dynamic equilibrium with the flipped-out state (Figure 8A, 8D). The equilibrium between the flip-in and flip-out states in the BMPP simulation can then explain the partial dehydration of the protonated phosphate group observed in the corresponding density profile (Figure 7B) and the GP results (Figure 6A). In the deprotonated BMP membrane, only the flipped-out state was observed throughout the simulation (Figures 8A, 8C), and therefore, the deprotonated phosphate group is continuously and fully exposed to the bulky solvent. The phosphate group is then observed as being fully hydrated in the BMPD density profile (Figure 7C). Of note, the number of hydrogen bonds (HBs) between the lipid polar head (phosphate group) and water molecules was calculated (a distance of 0.35 nm and 35° were taken as criteria). According to the results (Figure S12), there is a significant reduction in the number of HBs for BMPP-containing lipid bilayers in comparison with those observed for BMPD and DOPG bilayers. Such results also agree with those revealed by GP experimental values, reflecting the partial dehydration of the protonated BMP polar head due to the flipping-in state conformation.

**Figure 8:**
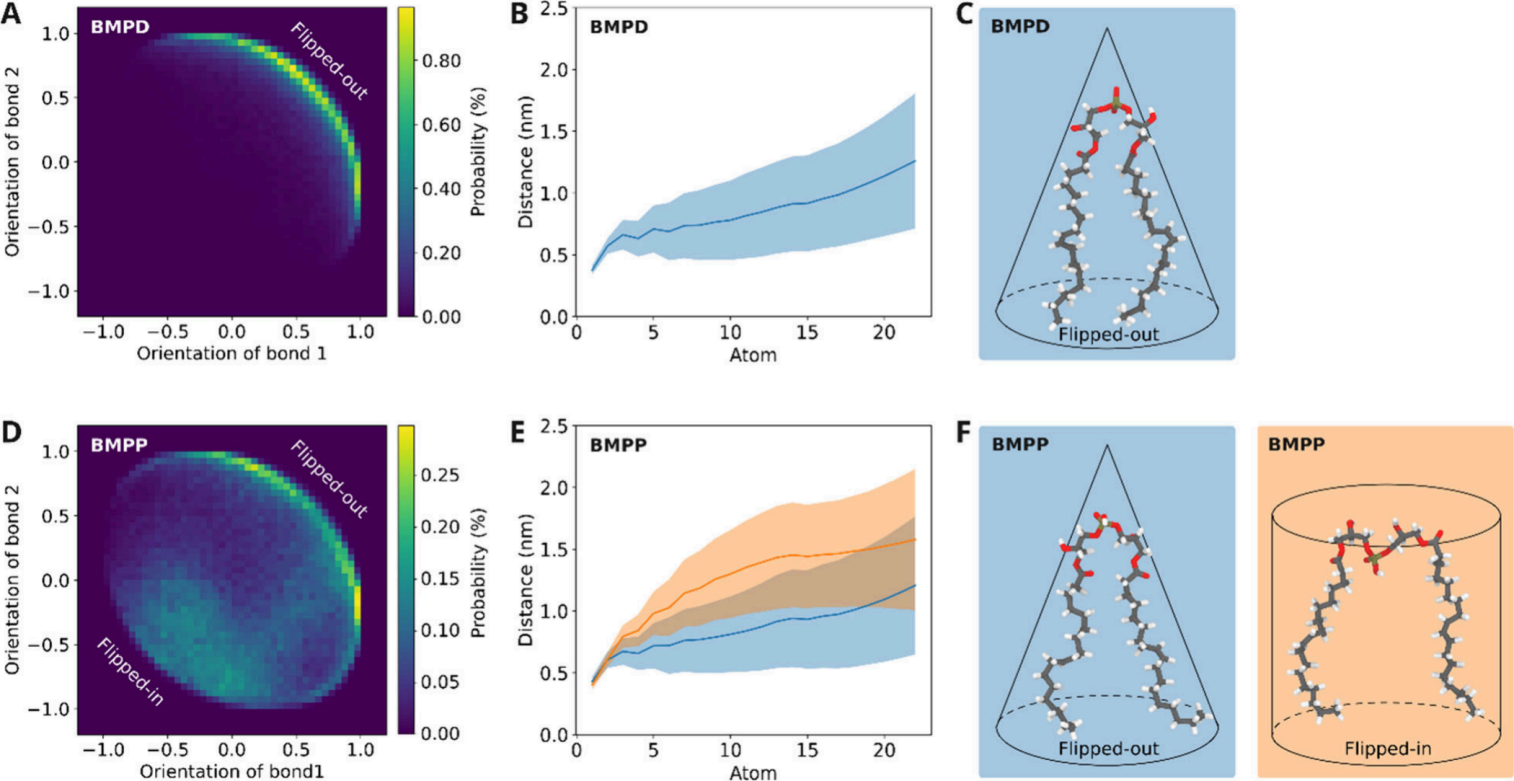
Conformational populations of the flipped-out and flipped-in states from MD simulations of deprotonated BMPD (neutral pH) and protonated BMPP (acidic pH). *Z*-component of the average distance between pairs of positions using as pairs the phosphorus and the carbon bonded oxygen atoms within the same phosphate group: (A) BMPD and (D) BMPP. Color scale indicates the occurrence of the pairs of positions. Average distance between opposite carbon atoms within pairs of acyl chains in the same BMP molecule: (B) BMPD and (E) BMPP. Representative structures from cluster analyses of populations of flipped-out and flipped-in conformations for (C) BMPD and (F) BMPP were generated by selecting the conformation with the smallest average RMSD from all sampled conformations within the most populated conformational cluster.

The conformational changes induced by the protonation of the BMP phosphate group were not restricted to the headgroup dynamics, but they also affected the acyl chain dynamics (Figures 8B, 8E). In the flipped-in state, the protonation of the BMP phosphate group under acidic conditions decreases the electrostatic repulsion among the negatively charged headgroups. Besides decreasing the average *A*_L_ and *V*_L_ (Table S2), BMP protonation allows the positioning of the hydroxyl group between the two acyl chains in the flipped-in conformation (Figure 8F). In this state, there is an increase in the distance between the two acyl chains within the same lipid molecule, minimizing any steric hindrance between the swinging hydroxyl group and their ester and carbonyl groups. This distance increases slightly along the carbon chain (Figure 8E), but the overall structure adopted by the flipped-in conformation of BMP has a cylinder-like shape (Figure 8F). On the other hand, in the flipped-out state, the acyl chain–pair distance increases more sharply toward the terminus region of the chain (Figure 8D, 8E), resulting in its conformations with a more conical-like shape (Figure 8C, 8F).

The MD simulations thus suggest that pH changes may shift the dynamical equilibrium between different conformational populations of BMP (Figure 8). The two main conformations correspond to distinct geometrical shapes, i.e., conical- and cylindrical-like, which in turn favor different membrane curvatures. At neutral pH, the flipped-out conformation is the predominant state of BMP. As the pH decreases, BMP molecules become increasingly protonated, first in the outer leaflet and then in the inner leaflet. This transformation favors the flipped-in state of BMP which will initially coexist with the flipped-out state favored by the deprotonated state of BMP (Figures 8D–F). Therefore, we hypothesize that the coexistence and interconversion of conical and cylindrical shapes of BMP as the pH is changed can promote local curvature frustration within the bilayer, driving the formation of undulations or folds observed in the GUVs. Interestingly, even when only about 10% of BMP lipids are protonated in our experimental assays at pH 4.5 (Figure S7) clear alterations in bilayer organization are observed. Such sensitivity suggests that the membrane’s collective properties respond nonlinearly to small variations in charge density: partial protonation is sufficient to shift the balance between electrostatic repulsion, headgroup hydration, and curvature stress, leading to smaller vesicles observed by DLS, and folded membranes over some parts of the membrane surface as revealed by GUV images and SAXS experiments.

## Conclusions

In this work, we investigated protein-free model lipid bilayers using a combination of experimental techniques and molecular dynamics simulations. Giant unilamellar vesicles composed of DOPG and 3,3′-BMP were examined at neutral and acidic pH, revealing that only BMP membranes at low pH develop extensive surface heterogeneity including interconnected vesicular protrusions indicative of local membrane folding. SAXS measurements support this observation, showing that BMP LUVs at pH 4.5 consist of a mixture of unilamellar vesicles and weakly correlated stacks of 2–3 bilayers. Despite these morphological differences, the electron density profiles of the BMP and DOPG bilayers at both pH values remain similar and are consistent with MD-derived profiles. Under acidic conditions, partial protonation of BMP reduces the membrane ζ-potential, reflecting diminished electrostatic repulsion between headgroups. This change leads to a decrease in area per lipid, as confirmed by Langmuir monolayers and MD simulations, and to reduced hydration at the polar–apolar interface, as detected by Laurdan GP measurements and supported by increases in acyl-chain order parameters.

MD simulations further show that protonation enables the phosphate group of BMP to adopt two distinct orientations: an outward-facing “flipped-out” conformation and an inward-facing “flipped-in” conformation penetrating the hydrophobic region. A dynamic equilibrium between these states exists only in protonated BMP, whereas the deprotonated lipid remains exclusively flipped-out. These conformations correspond to different effective molecular shapes, more cylindrical for flipped-in and more conical for flipped-out, which influence local curvature preferences when both states coexist at low pH.

Together, our results indicate that the combination of tighter packing in protonated BMP and the pH-dependent interconversion of headgroup orientations contributes to the curvature fluctuations and the membrane folding features observed experimentally. Importantly, substantial structural reorganization occurs even when only a small fraction (∼10%) of BMP molecules are protonated. Overall, this work highlights how variations in the charge state, hydration, and molecular geometry collectively dictate the physicochemical response of BMP-containing bilayers to acidic environments.

## Supplementary Material



## Notes

Supporting data for this study, including supplementary tables, GUV images, DLS measurements, SAXS curves, Langmuir isotherms, and molecular dynamics simulation results (PDF)

## ABBREVIATIONS

BMP: bis­(monoacylglycero)­phosphate
DLS: dynamic light scattering
DOPG: 1,2-dioleoyl-*sn*-glycero-3-phospho-(1′-rac-glycerol)
GP: generalized polarization
GUVs: giant unilamellar vesicles
LUVs: large unilamellar vesicles
MCT: modified Caillé theory
MD: molecular dynamics
MLVs: multilamellar vesicles
SAXS: small-angle X-ray scattering
