# Supplementary material for: Acidic pH Modulates Headgroup Orientation and Packing in Bis(monoacylglycero)phosphate Bilayers
Source: ACS Phys Chem Au. 2026 Mar 10;6(3):483–92. doi: 10.1021/acsphyschemau.5c00150 (PMC13220215; doi:10.1021/acsphyschemau.5c00150)
Supplement: Supplementary file 1 [file pg5c00150_si_001.pdf]

Supporting Information

Acidic pH Modulates Headgroup Orientation and Packing in Bis(monoacylglycero)phosphate Bilayers

Tayana Mazin Tsubone[a,b*], *Pedro Nunes de Oliveira Junior*[a, §], *Gustavo Scanavachi*[a,c,d], *Vinicius Firmino dos Santos*[e, §], *Antonio Rodrigues da Cunha*[a,f], *Ana Paula Ramos*[e], *Thereza A. Soares*[e,g], *Rosangela Itri*[a*]

[a]Departamento de Física Aplicada, Instituto de Física, Universidade de São Paulo, São Paulo, SP, 05508090, Brazil;

[b]Instituto de Química, Universidade Federal de Uberlândia, Uberlândia, MG, 38400-902, Brazil;

[c]Department of Pediatrics, Harvard Medical School, Boston, MA, 02115, USA;

[d]Program in Cellular and Molecular Medicine (PCMM), Boston Children's Hospital, Boston, MA, 02115, USA;

[e]Department of Chemistry, Faculty of Philosophy, Science, and Letters, University of São Paulo, Ribeirão Preto, SP, 14040-901, Brazil;

[f]Centro de Ciências de Balsas, Universidade Federal do Maranhão, Balsas, Maranhão, 65800-000, Brazil;

[g]Hylleraas Centre for Quantum Molecular Sciences, University of Oslo, Oslo, 0315 Norway.

* Corresponding authors: tayana.tsubone@ufu.br and itri@if.usp.br

[§]These authors contributed equally.

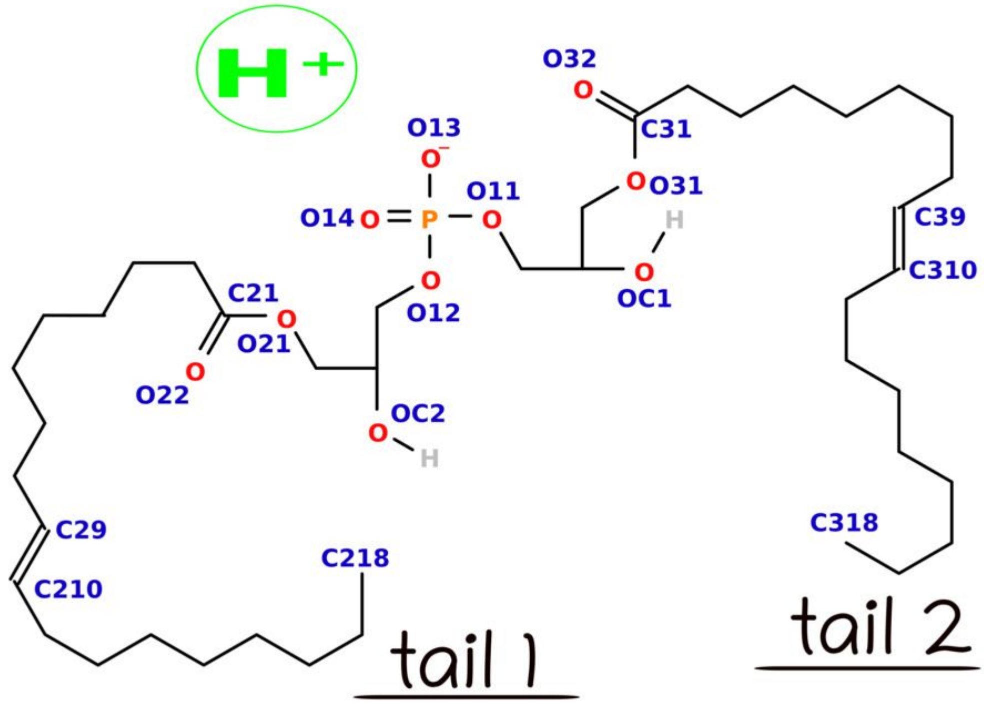

Figure S1. Structure of BMP (bis(monoacylglycerol)phosphate) with assigned atoms adopted throughout the molecular dynamic simulation.

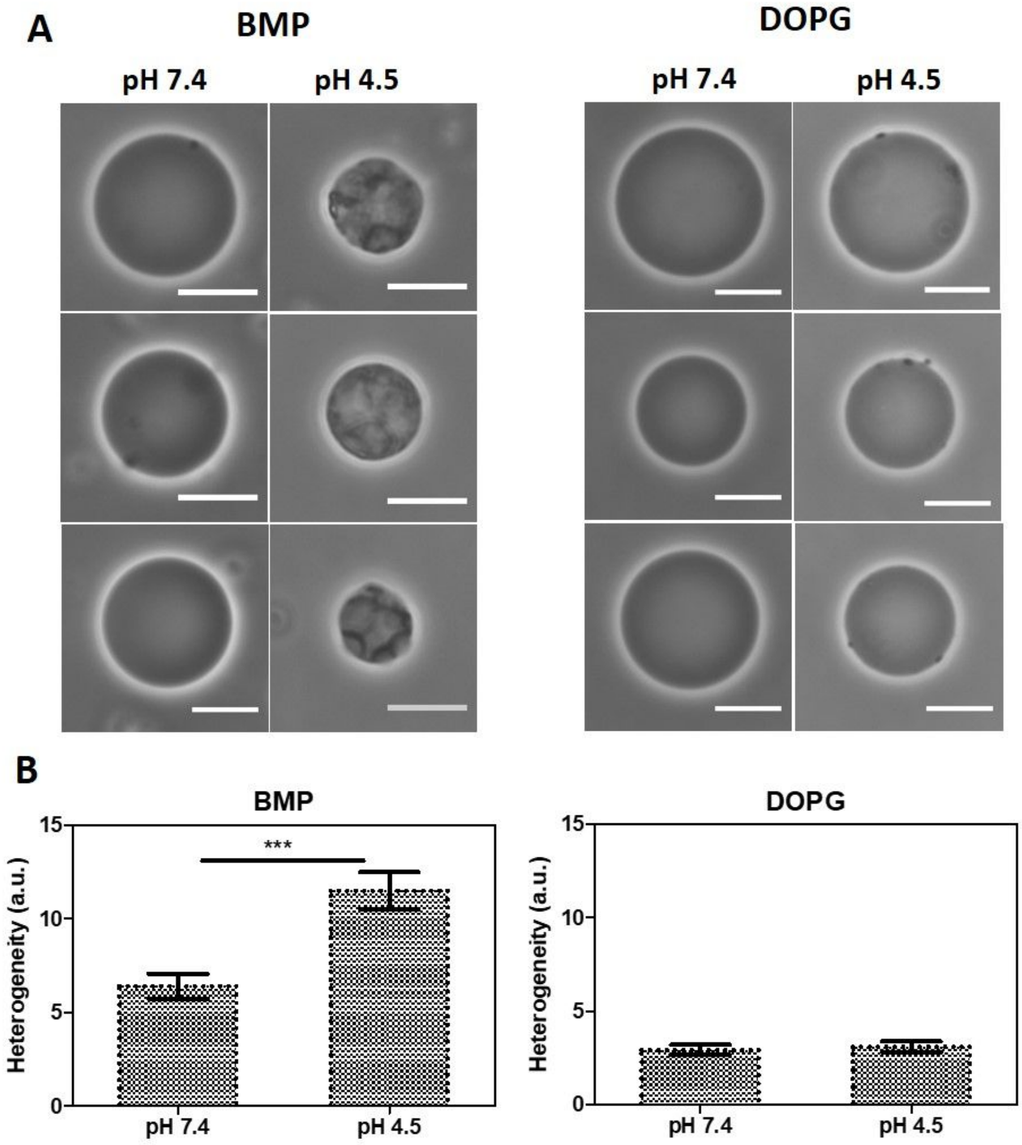

Figure S2. (A) Phase contrast microscopy of 3 representative images of GUVs composed of DOPG or BMP at 5 mM HEPES buffer pH 7.4 and 5 mM sodium acetate pH 4.5. (B) Heterogeneity of spherical vesicles measured by PIPA software (see supplemental material) from at least 15 GUVs. Bars represent means ± SEM and ***$p < 0.0001$.

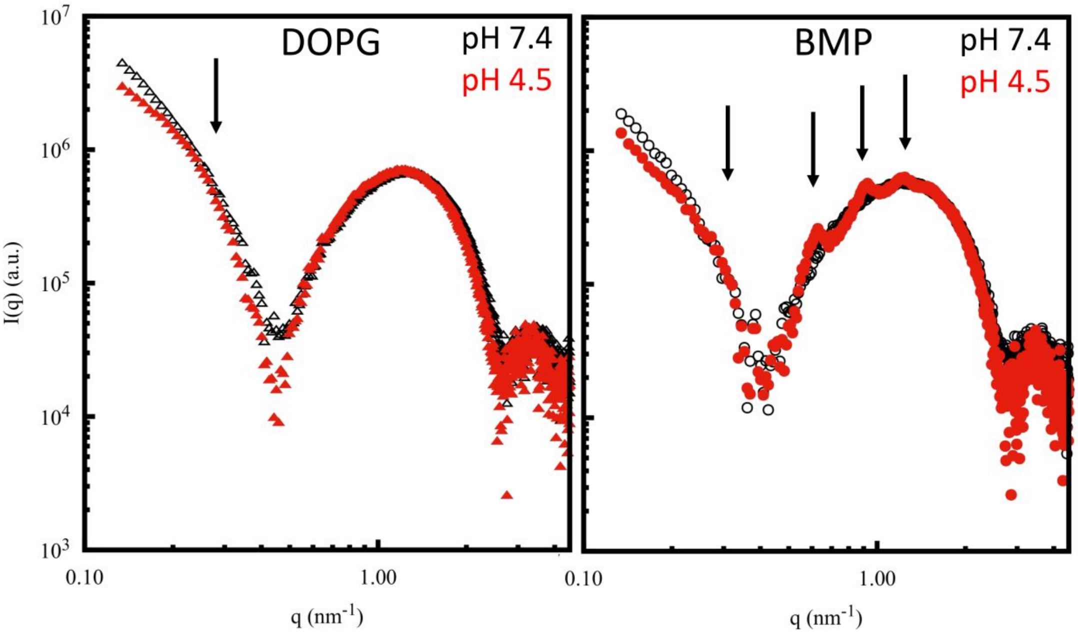

Figure S3. SAXS curves of LUVs composed of 10 mM DOPG and 10 mM BMP at pH 7.4 (5mM HEPES buffer – black open symbols) and at pH 4.5 (5mM acetate buffer – red full symbols) at 23ºC. The arrow in the DOPG graph indicates the bump at q ~ 0.24 nm^{-1} for the DOPG at pH 4.5. The first arrow in the BMP graphs indicates the bump at q ~ 0.30 nm^{-1} for the BMP at pH 4.5 and subsequent peaks for the same experimental data.

	pH 7.4		pH 4.5	
	**DOPG**	**BMP**	**DOPG**	**BMP**
R_{pol} (nm)	1.42(5)	1.28(5)	1.30(3)	1.26(5)
R_{CH2} (nm)	0.71(3)	0.81(3)	1.07(3)	0.99(5)
R_{CH3} (nm)	0.35(3)	0.28(3)	0.10(9)	0.10(7)
ρ_{pol}(e nm^{-3})	418(4)	420(4)	424(3)	419(3)
ρ_{CH2} (e nm^{-3})	258(4)	259(3)	259(3)	253(3)
ρ_{CH3} (e nm^{-3})	233(4)	219(3)	216(4)	211(4)
% of Multi			33(10)	26(10)
N			2(2)	3(3)
D (nm)			26.5(8)	20.6(8)
**nCaille**			**0.27(6)**	**0.06(2)**

Table S1. Parameters obtained from the fittings of lipid bilayer cross-section $P_t(q)$ Form Factors (Equation 2) to the experimental SAXS data (Figure 3) at 23 °C, using Genfit software [17]. Electron density and thickness for each region: polar headgroup (ρ_{pol}, R_{pol}), paraffinic chains (ρ_{CH2}, R_{CH2}), and methyl group (ρ_{CH3}, R_{CH3}). % of multi represents the % of multilamellar system coexisting with unilamellar vesicles. The uncertainty (in parenthesis) for each parameter was obtained by fitting the model to the same experimental curve 100 times.

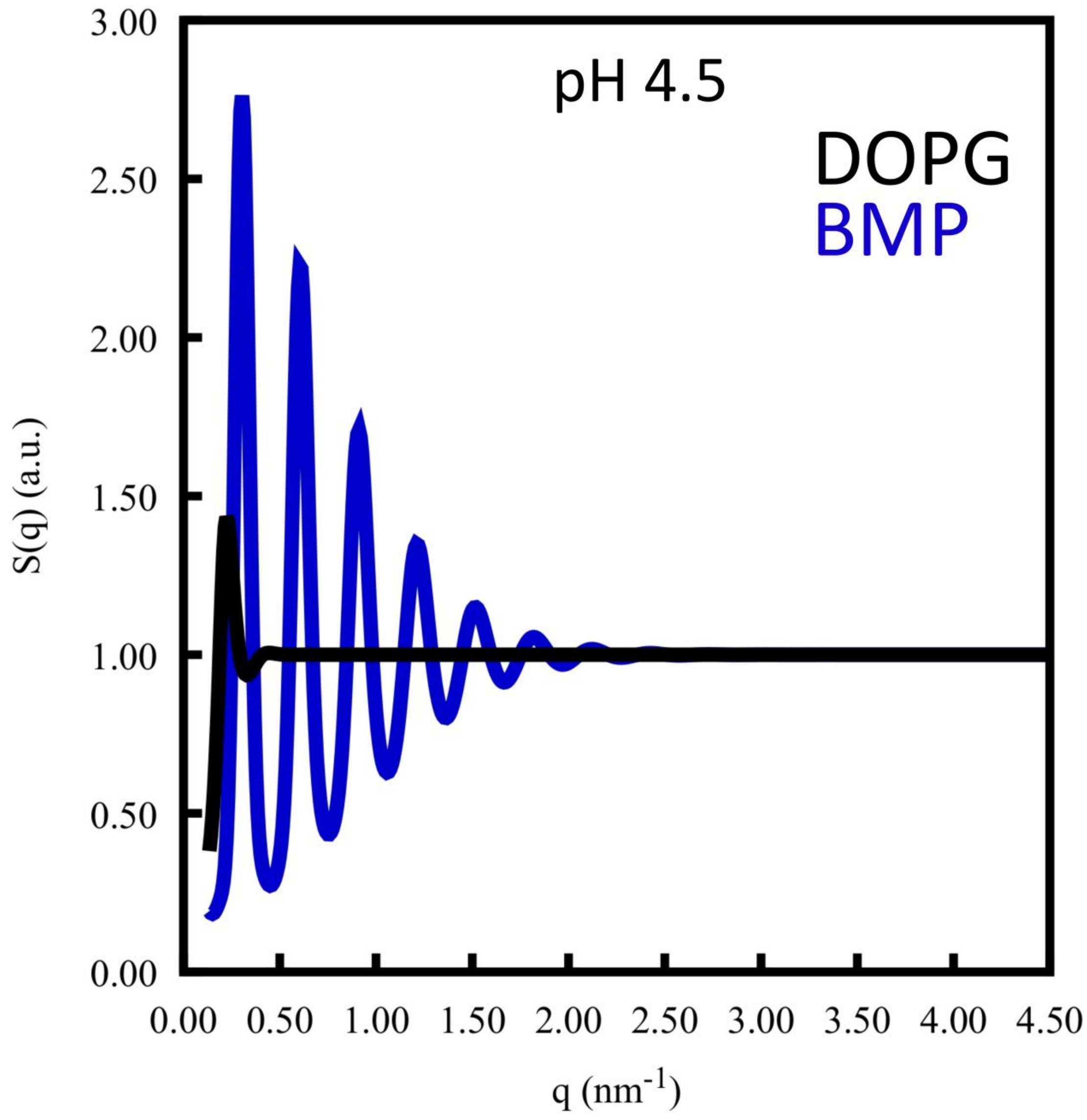

Figure S4. Structure factor, S(q), obtained by fitting Eq 2 to the SAXS experimental data from 10 mM DOPG (black) and 10 mM BMP (blue) at pH 4.5 (5 mM acetate buffer) using the modified Caillé theory (MCT).

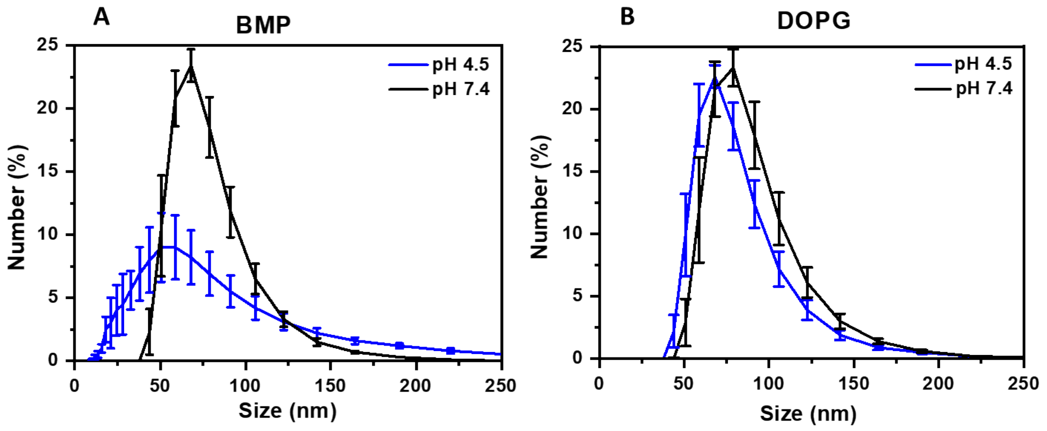

Figure S5. Distribution of LUVs sizes obtained by DLS measurements at 25ºC for (A) BMP and (B) DOPG LUVs at pH 4.5 (blue line) and pH 7.4 (black line). Vesicles mechanically passed through extrusion membranes with 100 nm pores. Error bars correspond to ± SEM from sample measurements.

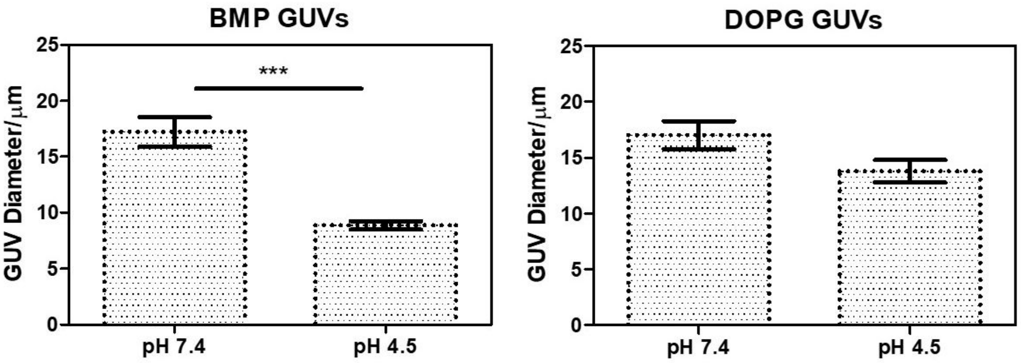

Figure S6. Average size of GUVs calculated by a home-made software from at least 15 GUVs. All GUVs are composed of 0.75 mM BMP (left) or 0.75 mM DOPG (right) at pH 7.4 (5 mM HEPES buffer) and pH 4.5 (5 mM acetate buffer). Bars represent means ±SEM, ***$p < 0.0001$ and *$p<0.05$.

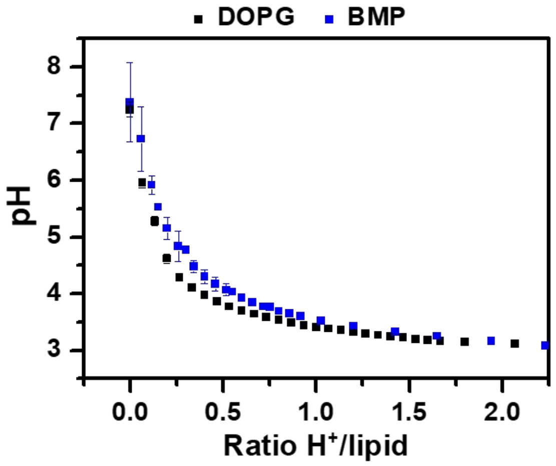

Figure S7. pH as a function of molar ratio (H^+/Lipid) for DOPG (■) and BMP (■) with the addition of HCl (0.01 M). Each square (■ or ■) corresponds to mean values and uncertainties from two independent measurements.

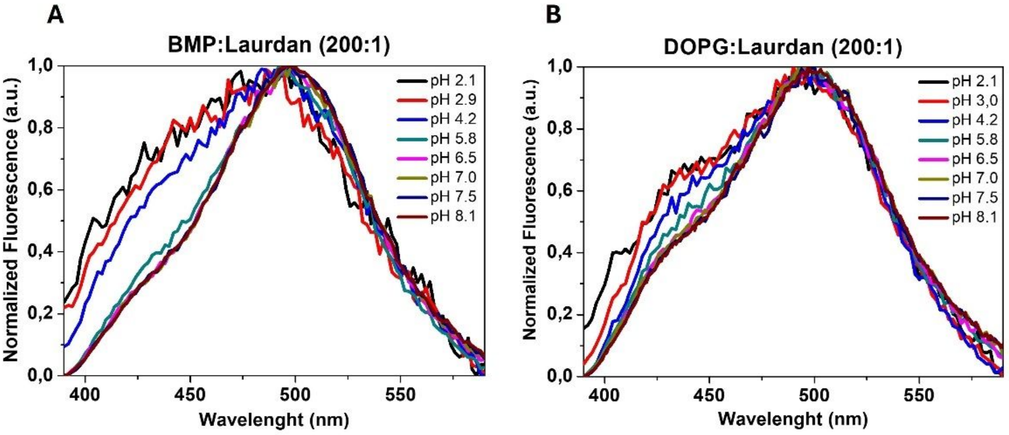

Figure S8. Normalized fluorescence emission of Laurdan spectra ($\lambda_{exc} = 355$ nm) recorded at different pH values (spectrum from top to bottom at pH 8.1, 7.5, 7.0, 6.5, 5.8, 4.2, 2.9 and 2.1) for the lipid systems: (A) BMP, (B) DOPG. All measurements were carried out at a lipid concentration of 0.1 mM and recorded at 25ºC. pH of MLVs samples was gradually lowered by adding aliquots of 100 mM HCl.

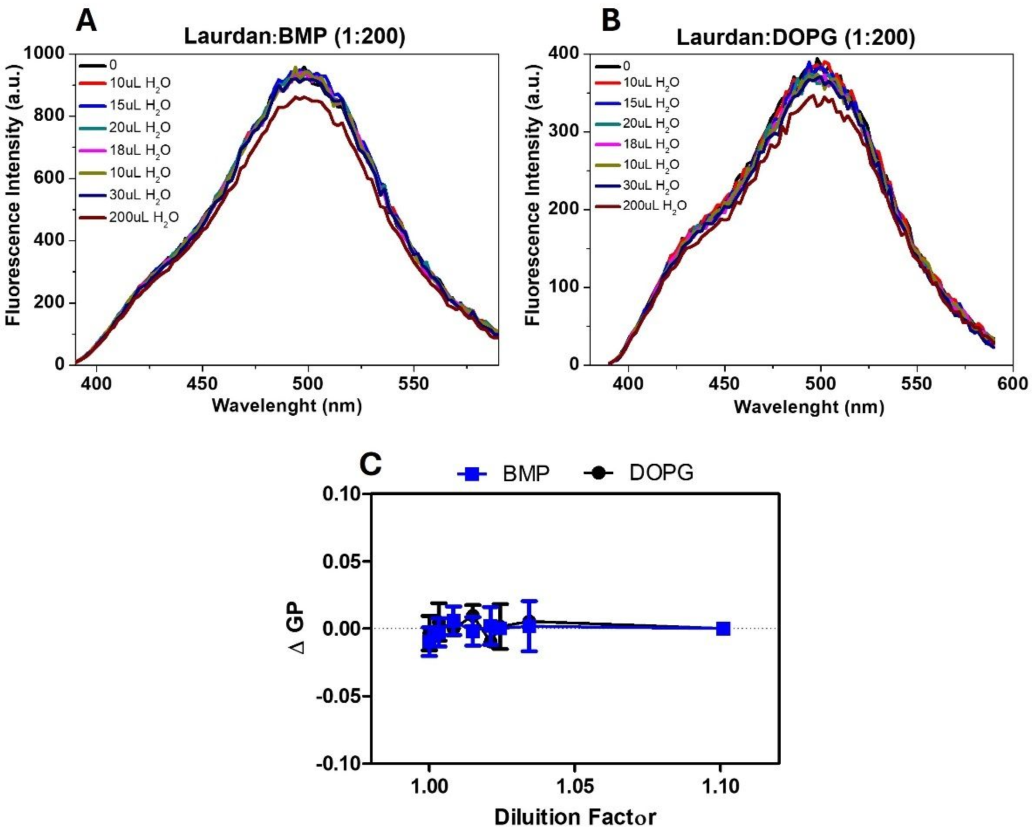

Figure S9. Laurdan fluorescence emission spectra (λ_{exc} = 355 nm) recorded at different dilution factors of water for (A) BMP and (B) DOPG membranes. (C) Calculated ΔGP values as a function of dilution factor for BMP (■) and DOPG (●) ΔGP is the difference between the GP value and its initial value at pH 8. Error bars correspond to ± SD from three sample measurements.

Langmuir monolayer investigation

Surface pressure-surface area (π-A) isotherms were assembled at room temperature of 23 ºC ± 1 ºC in a 216 cm^2 Langmuir trough (Insight-Brazil) to investigate differences in the lipids packing at the air-liquid interface. For this, 25 nM of lipid (BMP or DOPG) dissolved in chloroform were slowly dropped on either 5 mM sodium acetate buffer pH 4.5 or 5 mM HEPES buffer pH 7.4. The π-A isotherms were obtained at a compression rate of 0.42 $mm.s^{-1}$. Before the compression, 5 min were waited to guarantee solvent evaporation.

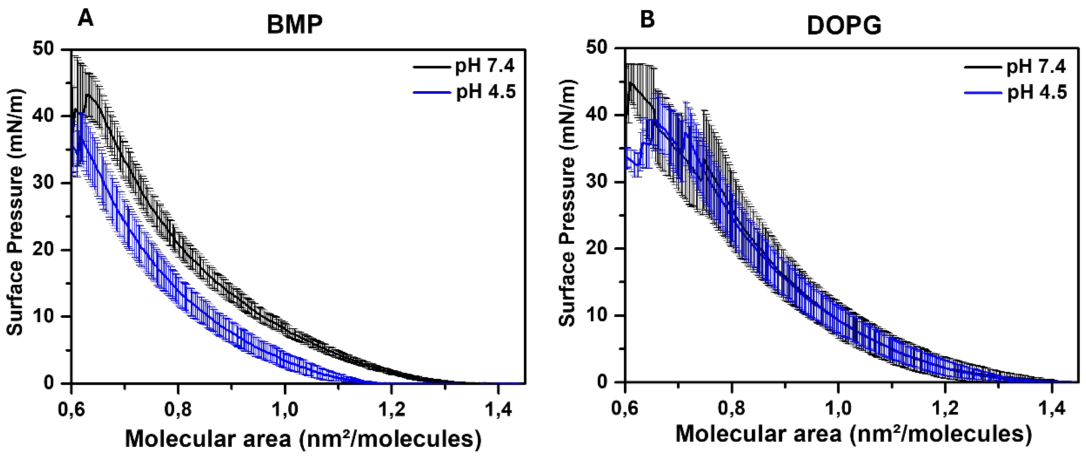

Figure S10. π-A isotherms of (A) BMP and (B) DOPG at pH 7.4 (black line) and pH 4.5 (blue line). Each π-A curve is represented by error bars of independent triplicate data.

Table S2. Average structural properties. Area per lipid, A_L nm^2, volume per lipid, V_L nm^3, membrane thickness, D_{HH} nm. Averages were calculated for the final 250 ns of simulation, and errors correspond to the standard deviation of uncorrelated data. Experimental values of A_L were obtained at the air-liquid interface via Langmuir monolayers at pH 7.4 and 4.5, π=30 mN/m and T=25 ºC.

**Systems**	**$\langle A_L \rangle_{calc}$**	**$\langle A_L \rangle_{exp}$**	**$\langle V_L \rangle$**	**$\langle D_{HH} \rangle$**
BMPD	0.758 (9)	0.721 (3)	1.290 (1)	3.37 (4)
BMPP	0.651 (6)	0.659 (3)	1.126 (7)	3.68 (4)
DOPG	0.714 (6)	0.762 (6)	1.323 (8)	3.43 (3)

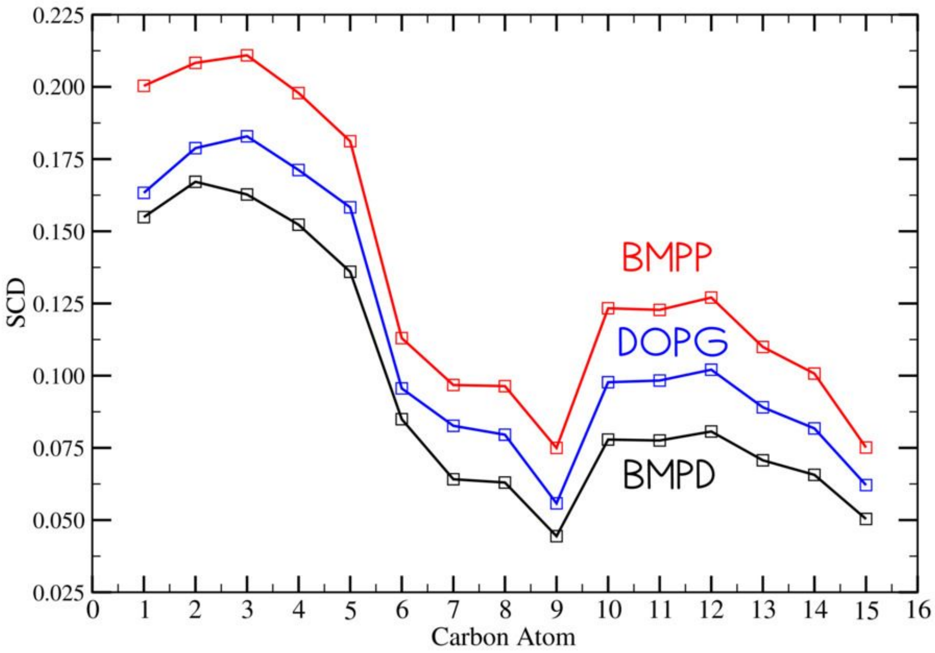

Figure S11. Deuterium order parameter, S_{CD}, calculates the relative orientation of the carbon−carbon bonds in the acyl chain with respect to the bilayer normal [44]. Carbon atom numbering starts with the carbonyl group. Simulated membranes are BMP in neutral (black) and acid (red) pH conditions, and DOPG (blue).

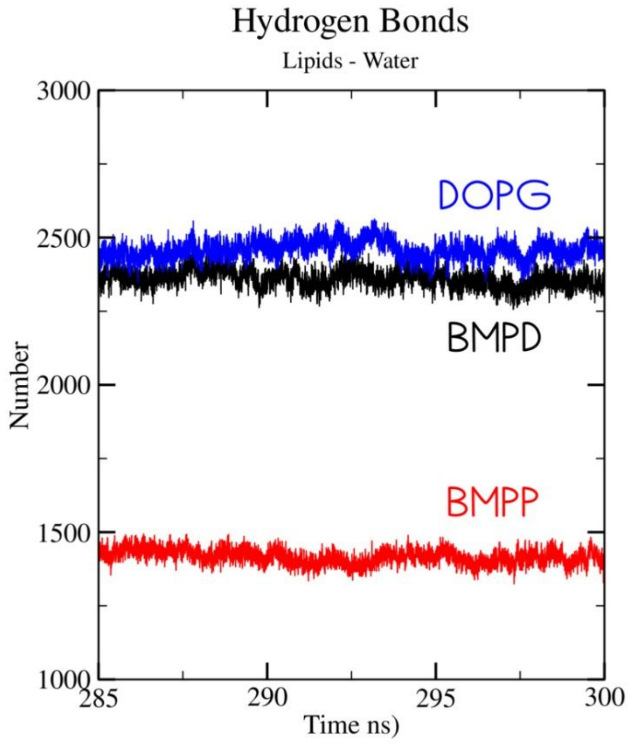

Figure S12. Number of hydrogen bonds (HBs) between lipids and water over the time in nanoseconds. Deprotonated BMPD (black); protonated BMPP (red); and DOPG (blue).

References

Spinozzi, F., Ferrero, C., Ortore, M. G., De Maria Antolinos, A., & Mariani, P. (2014). GENFIT: Software for the analysis of small-angle X-ray and neutron scattering data of macro-molecules in solution. *Journal of Applied Crystallography*, *47*(3), 1132–1139. https://doi.org/10.1107/S1600576714005147
